# Mycophenolic acid trough level assessment in patients with lupus nephritis; does it make a difference?

**DOI:** 10.1186/s12969-025-01074-7

**Published:** 2025-03-12

**Authors:** Ahmed E. Abdulgalil, Noha H. Elnagdy, Nehal M. Ramadan, Eman Hamza, Ayman Hammad, Mai S Korkor, Atef Elmougy, Ali Sobh, Marwa H Elnagdy

**Affiliations:** 1 Mansoura Nephrology and Dialysis Unit, Department of Internal Medicine, Faculty of Medicine, Mansoura University, Mansoura, Egypt; 2 Department of Rheumatology, Rehabilitation, and Physical Medicine, Faculty of Medicine, Mansoura University, Mansoura, Egypt; 3 Department of Clinical Pharmacology, Faculty of Medicine, Mansoura University, Mansoura, Egypt; 4 Medical Biochemistry and Molecular Biology, Faculty of Medicine, Mansoura University, Mansoura, Egypt; 5 Department of Pediatrics, Faculty of Medicine, Mansoura University Children’s Hospital, Mansoura University, Mansoura, Egypt

**Keywords:** Lupus nephritis, Mycophenolate mofetil, Mycophenolic acid trough level

## Abstract

**Introduction:**

Mycophenolate Mofetil (MMF) has become one of the cornerstone treatments of lupus nephritis (LN). It is converted into mycophenolic acid (MPA), an active metabolite, that displays high inter- and intra-individual pharmacokinetic variability. However, the routine monitoring of MPA trough level is still debatable.

**Objectives:**

The present study aims to evaluate the relationship between MPA trough levels and both clinical outcomes and drug-related adverse effects during the maintenance phase of LN in Egyptian patients.

**Methods:**

We included thirty-five adults and twenty-nine children with biopsy-proven class III and IV LN, who had been maintained on steroid and MMF as maintenance therapy for more than six months. Clinical and laboratory markers of lupus activity as well as MMF adverse events were reported. MPA trough levels were measured by High-Performance Liquid Chromatography (HPLC).

**Results:**

There was a significant association between low MPA trough levels and both flares and SLEDAI scores in the adult group (*P* = 0.027 and 0.019, respectively). Moreover, high MPA trough levels were associated with higher risk of gastritis in the same age group (*P* = 0.007). There was no significant association with any of the parameters studied in the pediatric group. Gastritis was the most frequent side effect in both age groups.

**Conclusion:**

MPA trough levels correlated with disease activity and gastritis in adult LN patients, and this may help to optimize MMF dosage in these patients. However, MPA concentration-effect relationships were not observed in pediatric patients.

**Supplementary Information:**

The online version contains supplementary material available at 10.1186/s12969-025-01074-7.

## Background

Systemic lupus erythematosus (SLE) is a chronic autoimmune disease that can affect all body systems [1]. Lupus nephritis (LN) represents one of the most serious SLE manifestations that occur in more than 50% of patients [2] and can eventually end with renal failure [3].

Lupus nephritis can be presented with hematuria, impaired renal function, new-onset hypertension and, or proteinuria that varies from mild to marked or nephrotic range proteinuria and nephrotic syndrome presentation [4]. Moreover, Interstitial nephritis and thrombotic microangiopathy can be seen among LN patients [5]. Renal biopsy remains the gold standard for diagnosis and classification of LN lesions to guide the plan of therapy. LN can be classified into six classes according to International Society of Nephrology-Renal Pathology Society 2003 classification system [6].

Treatment of LN includes induction of remission with steroids with either cyclophosphamide pulses or Mycophenolate Mofetil (MMF) followed by maintenance therapy with steroids combined with MMF or azathioprine. MMF has been shown to be as effective as cyclophosphamide in induction and more effective than azathioprine in maintenance phase [7, 8]. Moreover, a meta-analysis concluded that MMF can induce remission in severe cases of LN with lower risk of adverse effects that occur with cyclophosphamide pulses [9].

Monitoring of trough level of mycophenolic acid (MPA) could be beneficial in optimizing MMF doses as there are wide interindividual differences in drug pharmacokinetics [10], and this has been extensively studied in solid organ transplantation [11]. However, this may be affected by the differences in the clinical status of LN patients and the concomitant use of other immunosuppressive drugs. Previous literature has suggested an association between the level of MPA exposure and achievement of remission in LN [12]. Moreover, several studies have highlighted that MPA trough levels are correlated to MPA area under concentration-time (AUC) [13, 14]. In contrast, the impact of MPA trough level on lupus activity and drug-related side effects in patients with LN needs further evaluation [15]. Furthermore, studies are very limited in juvenile onset lupus. Hence, we conducted the present study on two groups of SLE patients: adults and children to assess the relationship between MPA trough level, disease activity, and drug adverse effects during the maintenance phase of LN treatment.

## Methods

### Patients and study design

The current study is a cross-sectional study that was conducted between June 2022 and December 2022. Adult cases (aged above eighteen years) were recruited from the Nephrology unit and the Rheumatology department in Mansoura University Hospital, while pediatric cases (**aged below 18 years**) were recruited from Mansoura University Children’s Hospital. The study protocol was approved by the Institutional Review Board of Mansoura University (R.21.11.1503). Informed consents were obtained from all included patients, or their legal guardians before enrolling in the study.

### Sample size calculation

The G*Power software (version 3.1.9.7) was used. Based on previous data available from, who reported a considerably large effect size (d = 1.000255) when comparing MPA trough value of two independent groups regarding acid-reflux symptoms [16]. A total sample size of 64 (distributed over the two groups in a ratio of 2.6) achieves 91% power to detect differences among the means using the Mann Whitney test with a 0.05 significance level.

### Inclusion and exclusion criteria

SLE patients diagnosed with class III or IV LN based on histopathological findings of renal biopsy, who were maintained on MMF therapy for more than six months were enrolled in the study. We excluded patients who were maintained on calcineurin inhibitors or receiving any drugs that could interfere with MPA pharmacokinetics as cholestyramine, acyclovir, or rifampicin.

#### Study outcomes

The primary outcome was to assess the correlation between the MPA trough levels, clinical parameters of lupus activity, SLEDAI scores and MMF related adverse effects during follow up of the included lupus nephritis patients.

### Clinical and laboratory assessment

All included patients were maintained on MMF therapy for more than six months before enrolling in the study. We started with a dose of 1 gm/day and titrated up to maximum of 2 gm/day according to lupus activity and SLEDAI scores.

Detailed history taking, and clinical examination were performed in all included cases with documentation of MMF related adverse effects as nausea, vomiting, diarrhea, or infections. Investigations were done for all included patients including CBC, CRP, ESR, serum creatinine, C3, C4, ANA, anti-dsDNA titers, urine analysis, 24-hour urinary protein collection, and MPA trough concentration. MPA trough levels between 2 and 4 µg/mL have been suggested to achieve maximum drug response with minimal side effects [17]. MPA trough concentration samples were obtained from patients on the same day as data collection and calculating activity scores. MPA trough levels samples were withdrawn 12 h after the last dose (C12). Patients were instructed to take MMF dosage every 12 h one month before taking samples. Activity scores were evaluated using Systemic Lupus Erythematosus Disease Activity Index 2000 (SLEDAI 2k) score (0 = no activity; 1 = mild activity with no therapeutic intervention; 2 = disease activity, but with improvement from previous visit; 3 = persistent activity/refractory to treatment; 4 = flare). Lupus flare means worsening of an already active system or a recent activity involving another system [18]. The most appropriate SLEDAI-2 K cut-off score to define lupus activity requiring drug adjustment is 3 or 4 [19].

### Plasma MPA trough level measurement

Plasma MPA trough levels were measured using the Reversed Phase High Performance Liquid Chromatography with Ultraviolet detection (RP-based HPLC-UV), according to the method previously described by Rissling et al. [20].

### Materials

MPA analytical standard (98.5% purity, 89287) was purchased from Sigma Aldrich, USA. Other chemicals and solvents were of HPLC purity grades and were purchased locally. Control samples were obtained from healthy volunteers.

### Sample preparation

For measurement of MPA C0, lithium-heparin blood samples were withdrawn from all included patients. To ensure standardization of MPA results, samples were collected 12 h after the last evening MMF dose (MPA C0). Then, centrifugation of samples was done for 10 min, then the plasma was collected and stored at -80 C.

### Instrument and HPLC method

The HPLC system consisted of a Waters 2690 Alliance HPLC system (Milford, USA) equipped with a Waters 996 photodiode array detector set on 214 nm, an autosampler tempered at RT, column oven at 55 C and compatible data processing software. The drug was separated by a C18 Inertsil ODS analytical column (150 mm×4.6 mm, particle size 5 μm; GL Sciences, Japan) equipped by a guard column of the same packing. The mobile phase was composed of acetonitrile– 50 mmol/L potassium dihydrogen phosphate, pH 2.4. (30:70; v/v) with a flow rate of 1.5 ml/min.

### Standard preparation and assay procedure

A stock solution of 1.0 mg/mL MPA in acetonitrile was prepared, from which seven calibration standards were prepared. The drug-free human plasma was used for serial dilutions of MPA stock solutions to obtain concentrations of 0.7, 0.6, 0.5, 0.4, 0.3, 0.2, 0.1 µg/ml.

Using the method previously described, a 125µL acetonitrile was added to each aliquot of fifty µL calibration standards or plasma samples. The mixture was well-mixed for 15 s then centrifuged in 1.5mL polypropylene tubes at 1500 g for 5 min. Then supernatant volume of 75µL were mixed with 50µL 50 mmol potassium dihydrogen phosphate, pH 2.4. After being filtered through 0.22 μm Nylon syringe filter, a volume of 20µL was injected. MPA peak was extrapolated (retention time: MPA peak, 3.30 min). The calibration curve was obtained (range, 0.1–0.7 µg/L) (Supplementary Fig. 1).

### Statistical analysis

We used SPSS version 25 software (Statistical Package for the Social Sciences) for tabulation and analysis of the data. Data were presented as mean and Standard Deviation (SD) or median and interquartile range (IQR) in quantitative data and number (N) and percent (%) in qualitative data. The Student T test and Mann Whitney Test (U test) were used to compare parametric quantitative variables and non-parametric variables, respectively. Spearman’s rank correlation analyses were performed to examine the unadjusted association between MPA C0 and other clinical variables with continuous data. Receiver-operated characteristic (ROC) curves were generated to determine the cut-off level of estimated MPA C0 related to different clinical outcomes. P-value < 0.05 is considered statistically significant.

## Results

The preset study included sixty-four patients with LN; thirty-five were adults (mean age of 30.53 years) and twenty-nine of them were children (mean age of 14.93 years). Patient characteristics, clinical findings, laboratory parameters, treatments, and MMF-related side effects are illustrated (Table 1). There was a female predominance in both age groups with a median SLEDAI of 5 in pediatrics (0–22) and nine in adults (0–30). All patients were maintained on steroids and MMF. MMF related side effects included gastritis, leucopenia, and infection. Gastritis was the most frequent one observed in 27.6% and 28.6% in pediatric and adult groups, respectively.

**Table 1:** Demographics, clinical findings, laboratory results, treatments and side effects observed in the study groups

	Pediatric group (*n* = 29)	Adult group (*n* = 35)
Age (years) mean ± SD	14.93 ± 1.79	30.54 ± 9.01
Sex:		
Male Female BMI median (min-max) Disease duration in month median (min-max)	8 (27.6%) 21 (72.6%) 22. 27 (14.22–45.2) 12 (6–30)	4 (11.4%) 31 (88.6%) 25.42 (18.61–33.06) 24 (6–84)
Clinical findings N (%)		
Hypertension Malar rash Oral ulcer Alopecia Photosensitivity Arthritis Myalgia Pleurisy Neurological symptoms Carditis LN class III LN class IV Flare	5 (17.2%) 7 (24.1%) 7 (24.1%) 7 (24.1%) 1 (3.4%) 1 (3.4%) 0 (0%) 0 (0%) 0 (0%) 0 (0%) 13(44.8%) 16 (55.2%) 13 (44.8%)	11 (31.4%) 10 (28.6%) 3 (8.6%) 14 (40%) 7 (20%) 10 (28.6%) 2 (5.7%) 3 (8.6%) 1 (2.9%) 3 (8.6%) 15 (42.9%) 20 (57.1%) 12 (34.3%)
Investigations		
Hemoglobin (g/dl) WBCs (10^3^/L) CRP (mg/dl) ESR (mm/hr) Creatinine (mg/dl) C3 (mg/dl) C4 (mg/dl) ANA dsDNA titers 24 h. urine protein (mg/day) Hematuria (cells/HPF)	11.6 (7.5–13.5) 8 (3.8–12) 2 (0–45) 35 (20–75) 0.6 (0.3–1.2) 90 (51–204) 10 (8–20) 5.5 (0–15) 1.1 (0–4) 350 (150–2700) 3 (1–60)	11 (7.2–14) 5 (3.1–12) 3(0–80) 35 (7-119) 0.8 (0.4–2.2) 100 (40–140) 12 (6–28) 2 (0-10.7) 1 (0–8) 540 (58-6500) 5 (0–20)
Treatment		
Steroid dose mg/d median(min-max)	20 (5–45)	20 (2.5–60)
HCQ no (%) Dose: 200 mg/d 400 mg/d	29 (100%) 29 (100%)	31 (88.6%) 5 (16.1%) 26 (83.9%)
MMF dose 1000 mg/d 1250 mg/d 1500 mg/d 2000 mg/d	25 (86.2%) 0 (0%) 1 (3.4%) 3 (10.3%)	9 (25.7%) 1 (2.9%) 8 (22.9%) 17 (48.5%)
MPA trough level (µg/mL)	0.7 (0.27–8.79)	0.83 (0.15–5.86)
MMF side effects N (%)		
Gastritis Leucopenia Infection	8 (27.6%) 1 (3.4%) 1 (3.4%)	10 (28.6%) 6 (17.1%) 4 (11.4%)
SLEDAI median (min-max)	5 (0–22)	9 (0–30)

Quantitative data are presented as median ± range, unless otherwise stated. Categorical variables and adverse event data were presented as N and percentage (%), N: number, BMI: body mass index, LN: lupus nephritis, CRP: C reactive protein, ESR: erythrocyte sedimentation rate, C3: complement C3, dDNA: double stranded DNA, HCQ: hydroxy chloroquine, MMF: mycophenolate mofetil, MPA: mycophenolic acid, SLEDAI: SLE Disease Activity Index

**Fig. 1:**
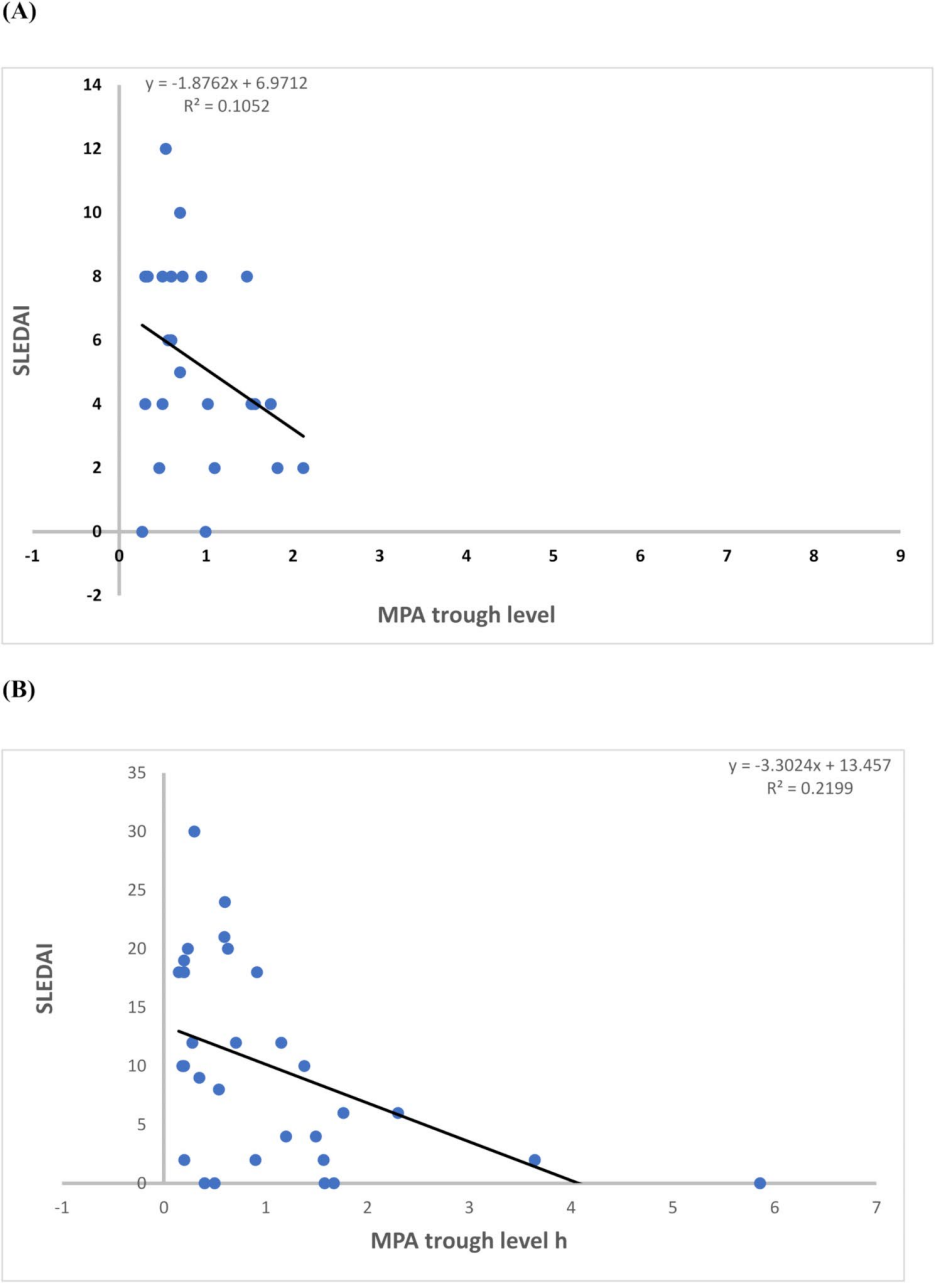
Spearman’s rank correlation between MPA trough concentrations (µg/mL) and SLEDAI in the pediatric (**A**) and Adult (**B**) groups

Regarding the correlation between the MPA trough levels and the clinical outcomes, significantly lower MPA trough levels were observed in the adult patients with lupus flare, compared to those in remission (*P* = 0.027) (Table 2). A significant, yet weak, negative correlation was also observed between MPA trough levels and SLEDAI in the adult group (*P* = 0.019) (Fig. 1). In addition, significantly higher MPA trough levels were observed in adult patients with gastritis compared to those without gastritis (*P* = 0.007). However, in the pediatric group, no significant association was found between MMF trough levels and any of the parameters studied (Table 3).

**Table 2:** Comparison between MPA trough levels (µg/mL) and clinical findings/MMF-related side effects in both study groups

		Pediatric group (*n* = 29)			Adult group (*n* = 35)		
		**№**	**MPA trough level**	**P-value**	**№**	**MPA trough level**	**P-value**
			mean ± SD			mean ± SD	
Clinical findings							
Malar rash:	Present	7	1.45 ± 2.44	0.221	10	1.03 ± 1.4	0.112
	Absent	22	1.43 ± 1.8		25	1.47 ± 1.4	
Oral ulcer:	Present	7	1.59 ± 2.4	0.683	3	2.52 ± 1.98	0.316
	Absent	22	1.38 ± 1.81		32	1.24 ± 1.31	
Alopecia	Present	7	1.59 ± 2.4	0.683	14	1.26 ± 1.37	0.533
	Absent	22	1.38 ± 1.81		21	1.41 ± 1.44	
Photosensitivity	Present	1	1.47	0.473	7	1.69 ± 1.48	0.248
	Absent	28	1.43 ± 1.96		28	1.26 ± 1.39	
Arthritis	Present	1	6.98	0.12	10	1.05 ± 1.36	0.139
	Absent	28	1.24 ± 1.63		25	1.47 ± 1.42	
Mayalgia	Present	0	-	-	2	2.07 ± 2.5	0.619
	Absent	29	1.43 ± 1.93		33	1.3 ± 1.35	
Pleurisy	Present	0	-	-	3	0.37 ± 0.21	0.133
	Absent	29	1.43 ± 1.93		32	1.44 ± 1.42	
Cerebritis	Present	0	-	-	1	0.6	0.8
	Absent	29	1.43 ± 1.93		34	1.37 ± 1.41	
Carditis	Present	0	-	-	3	0.37 ± 0.21	0.118
	Absent	29	1.43 ± 1.93		32	1.44 ± 1.42	
Flare	Present	13	1.25 ± 1.9	0.062	12	0.75 ± 0.92	*0.027
	Absent	16	1.59 ± 1.997		23	1.66 ± 1.51	
Side effects							
Gastritis	Present	8	1.65 ± 2.25	0.769	10	2.42 ± 1.76	*0.007
	Absent	21	1.35 ± 1.84		25	0.92 ± 0.96	
Leucopenia	Present	1	1.82	0.231	6	1.41 ± 1.69	0.526
	Absent	28	1.42 ± 1.96		29	1.34 ± 1.36	
Infection	Present	1	0.5	0.338	4	1.84 ± 1.39	0.213
	Absent	28	1.47 ± 1.95		31	1.28 ± 1.4	

MMF trough levels are presented as mean ± SD and compared by Mann Whitney U-test. №: number, MPA: mycophenolic acid

**Table 3:** Correlations of MPA trough concentrations with laboratory findings and SLEDAI in study groups

	Pediatric group (*n* = 29)		Adult group (*n* = 35)	
	**Correlation Coefficient**	**Sig. (2-tailed)**	**Correlation Coefficient**	**Sig. (2-tailed)**
MMF dose	0.211	0.272	0.283	0.099
Hemoglobin	-0.043	0.825	-0.079	0.651
Leucocytes	-0.216	0.261	0.093	0.594
CRP	-0.295	0.121	0.278	0.106
ESR	0.059	0.761	-0.026	0.884
Proteinuria	-0.154	0.425	-0.016	0.926
Hematuria	0.007	0.971	-0.206	0.234
Creatinine	0.394	0.035	0.1	0.566
C3	-0.036	0.854	0.196	0.227
C4			0.204	0.216
ANA	-0.019	0.921	-0.141	0.42
dsDNA titre	-0.104	0.591	-0.184	3870.289
SLEDAI	-0.274	0.15	-0.393	**0.019**

Spearman’s rank correlation analyses were used with a P-value of < 0.05 considered statistically significant. MMF: mycophenolic mofetil, CRP: C reactive protein, ESR: erythrocyte sedimentation rate, C3: complement C3, dDNA: double stranded D

**Fig. 2:**
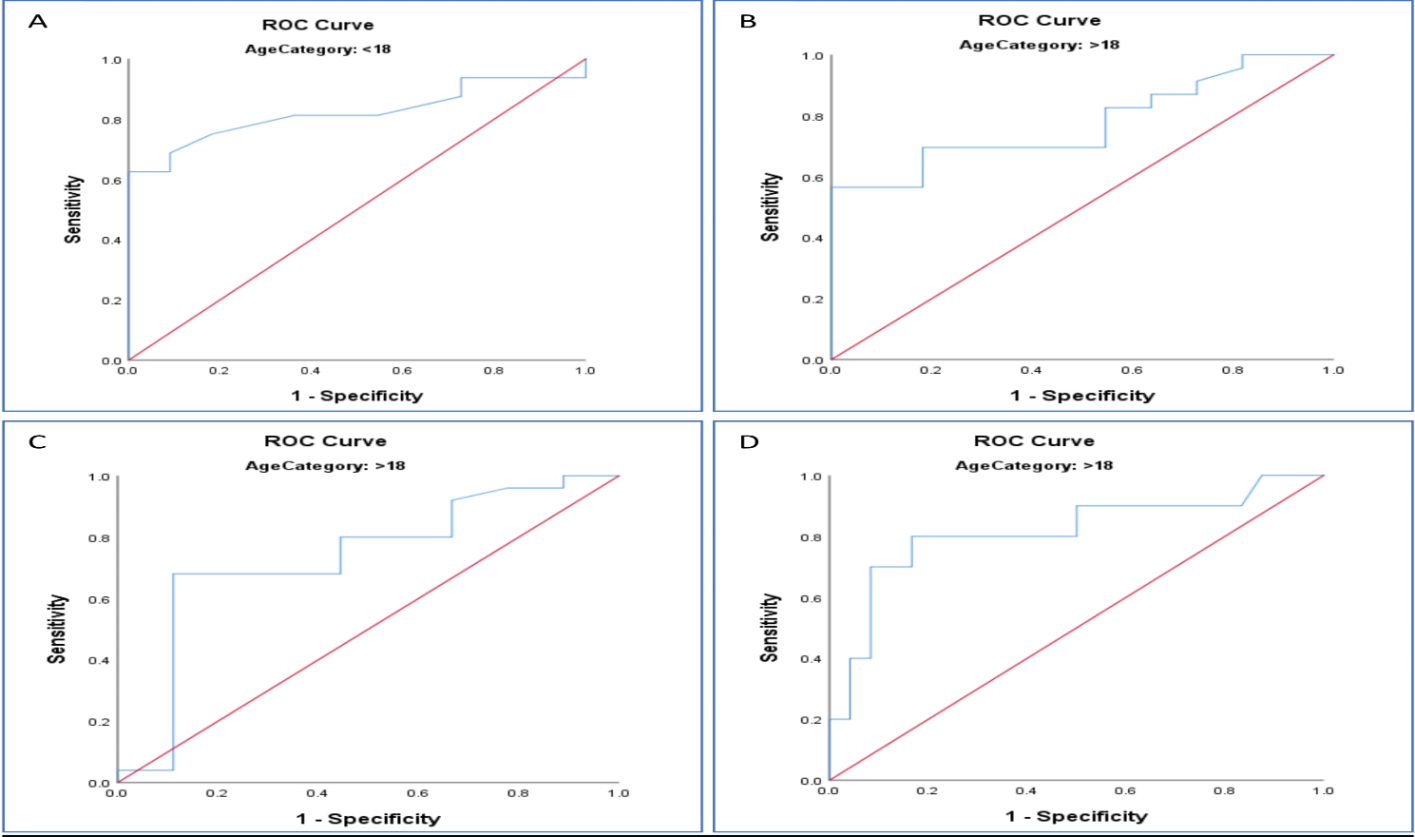
Receiver operating characteristic (ROC) curves of MPA trough concentration to predict therapeutic outcomes of MFF treatment. The fraction of true-positive (sensitivity) and that of false-positive results (1-specificity) for flare in pediatrics (**A**), flare in adults (**B**), malar rash in adults (**C**), gastritis in adults (**D**). For area under the curve of ROC curves and 95% confidence intervals, please refer to supplementary Fig. 2. A value of 0.5 (reference red line) is no better than by chance and a value of 1.0 reflects a perfect indicator

Regarding the therapeutic response to MMF, ROC curves detected that the cut-off levels of MPA trough concentrations below which flare is increasingly encountered. In adults, MPA troughs lower than or equal to 0.67 µg/mL were proposed to be associated with higher risk of flares (70% sensitivity and 82% specificity, p value < 0.01). while in pediatric group, trough level below 0.716 µg/mL (69% sensitivity and 91% specificity, p value < 0.005). Moreover, in adults, malar rash was more frequent with MPA trough levels lower than 0.67 µg/mL (68% sensitivity and 93% specificity, *p* = 0.042). On the other hand, gastritis was increasingly seen with MPA trough levels equal to or greater than 1.29 µg/mL (80% sensitivity and 83% specificity, p value = 0.004) (Fig. 2 and supplementary Fig. 2).

## Discussion

Although MMF represents a cornerstone treatment for LN, the optimal dose of MMF and target MPA level to maximize efficacy and minimize toxicity is still debatable [3]. MMF under dosage may lead to uncontrolled disease activity, however MPA over exposure is costly and can lead to serious complications such as recurrent infections and bone marrow depression [15]. Additionally, there is wide inter-patient variability in MPA pharmacokinetics, Hence, MPA therapeutic drug monitoring (TDM) is essential for appropriate MMF dosage [14, 21].

Inter-individual variability is less significant in patients suffering from autoimmune disease (AID) than those with kidney transplantation. This is due to less fluctuation in concomitant therapy in AID patients. Thus, a single MMF trough-level seems to be a worthwhile monitoring approach in those with AID [13].

The most widely used methods for TDM of MPA are either AUC or trough levels monitoring [21]. There have been trials to substitute the standard AUC measurement with single time-point MPA trough levels. Studies showed that MPA-AUC had significant positive correlations with both MPA trough levels and MMF therapeutic responses. Although C1, C2, and C12 MPA levels all correlate well with AUC0–12, MPA C12 trough level remains an attractive and practical tool to guide MMF therapy [13, 15].

The present study revealed that, in adult group, MPA trough levels had significant negative correlation with the occurrence of lupus flare and the level of disease activity as indicated by SLEDAI 2k score (P values 0.027 and 0.019, respectively). Similarly, Yap et al. concluded that lower MMF trough levels increased the risk of renal flares and higher trough levels increased incidence of anemia, decreased levels of immunoglobulin and increased risk of serious infections [15].

Moreover, other studies reported that lower MPA trough levels increased the risk of disease recurrence, whereas higher levels led to more MMF-related adverse effects such as gastrointestinal complaints (*P* = 0.007) [13], hematological complications and repeated infections [22].

In agreement with Yap et al. MPA trough levels did not correlate with the levels of anti-dsDNA or C3 [15]. This can be attributed to the effect of concomitant steroids and variable response of various components of the immune system.

The use of TDM to optimize the MMF treatment in children is not widely adopted except in solid organ transplantation [23]. There were only very few studies done on children with SLE [24]. Regarding pediatric patients, MPA trough levels did not correlate with either flare, disease activity or occurrence of MMF related-side effects. This may be explained by the fact that ideal dosing of MMF in children with LN is not yet well described and that weight- or body surface area-based MMF dosage does not predict MPA pharmacokinetics or dynamics [24].

The developmental changes (ontogeny) in physiological and biochemical parameters in children should be considered during TDM as they may influence the drug absorption, body fluid distribution, metabolism, and finally the clearance from the body. TDM should be used to improve therapeutic outcomes, by improving drug efficacy and/or decreasing adverse effects [25]. Data from the few studies performed on pediatric LN patients suggested that personalized MPA-AUC based MMF dosage might be safer and more cost-effective than the currently used body surface area-based approach [26].

However, MPA-AUC monitoring is laborious, not suitable in daily practice and demands multiple blood sampling. Thus, it is time and resource-consuming, and not well tolerated by children [25]. Additionally, data regarding its use in pediatrics is conflicting. Higher MPA-AUC was associated with increased risk of bone marrow depression, severe leukopenia [27] and recurrent infections [28]. Moreover, another study concluded that the clinical response six months after MMF initiation is highly dependent on the initial MPA-AUC values, and they found that MPA-AUC of 30 mg/l or higher led to better control of lupus activity [29]. In contrast, no significant associations were found between MPA-AUC and MMF related side effects in other studies [30, 31]. Indeed, yet there is no adequate evidence suggesting the best modality for cost-effective MMF dosage in children with lupus.

The limitations of the current study include the relatively small sample size and lack of MPA trough levels and AUC correlation. Future randomized controlled detailed pharmacokinetic trials with large sample size and prolonged follow up are needed to ascertain the correlation of MPA trough level and LN activity and/or MMF adverse effects. Also, further studies are still needed in LN pediatric patients to clarify the reasons for the missing correlation between MPA trough level, disease activity and MMF side effects.

## Conclusion

MPA trough concentrations correlated with disease activity and gastritis in adult LN patients, and this may help to optimize MMF dosage without causing serious adverse effects. However, this MPA trough concentration-effect relationship was not evident in pediatric patients that requires more extensive evaluation in future studies.

## Electronic supplementary material

Below is the link to the electronic supplementary material.

Supplementary Material 1

## Data Availability

No datasets were generated or analysed during the current study.

## Abbreviations

LN: Lupus Nephritis
SLE: Systemic Lupus Erythematosus
MMF: Mycophenolate Mofetil
MPA: Mycophenolic Acid
SLEDAI 2K score: Systemic Lupus Erythematosus Disease Activity Index
TDM: Therapeutic Drug Monitoring
AID: Autoimmune Disease

## References

[1] Godron-Dubrasquet A, Woillard J-B, Decramer S, Fila M, Guigonis V, Tellier S, et al. Mycophenolic acid area under the concentration-time curve is associated with therapeutic response in childhood-onset lupus nephritis. Pediatr Nephrol. 2021;36:341–7. 32856157 10.1007/s00467-020-04733-x

[2] Feldman CH, Hiraki LT, Liu J, Fischer MA, Solomon DH, Alarcón GS, et al. Epidemiology and sociodemographics of systemic lupus erythematosus and lupus nephritis among US adults with Medicaid coverage, 2000–2004. Arthr Rhuem. 2013;65(3):753–63. 10.1002/art.37795 PMC3733212 23203603

[3] Mok CC, Yap DYH, Navarra SV, Liu Z, Mh Z, Lu L, et al. Overview of lupus nephritis management guidelines and perspective from a sia. Nephrology. 2014;19(1):11–20. 23876069 10.1111/nep.12136

[4] Ameer MA, Chaudhry H, Mushtaq J, Khan OS, Babar M, Hashim T et al. An overview of systemic lupus erythematosus (SLE) pathogenesis, classification, and management. Cureus. 2022;14(10). 10.7759/cureus.30330 PMC9662848 36407159

[5] Aringer M, Smolen JS. Cytokine expression in lupus kidneys. Lupus. 2005;14(1):13–8. 15732282 10.1191/0961203305lu2053oa

[6] Park MH. International Society of Nephrology/Renal Pathology Society 2003 Classification of Lupus Nephritis. J Pathol Translational Med. 2006;40(3):165–75.

[7] Henderson LK, Masson P, Craig JC, Roberts MA, Flanc RS, Strippoli GFM, et al. Induction and maintenance treatment of proliferative lupus nephritis: a meta-analysis of randomized controlled trials. Am J Kidney Dis. 2013;61(1):74–87. 23182601 10.1053/j.ajkd.2012.08.041

[8] Palmer SC, Tunnicliffe DJ, Singh-Grewal D, Mavridis D, Tonelli M, Johnson DW, et al. Induction and maintenance immunosuppression treatment of proliferative lupus nephritis: a network meta-analysis of randomized trials. Am J Kidney Dis. 2017;70(3):324–36. 28233655 10.1053/j.ajkd.2016.12.008

[9] Zhu B, Chen N, Lin Y, Ren H, Zhang W, Wang W, et al. Mycophenolate mofetil in induction and maintenance therapy of severe lupus nephritis: a meta-analysis of randomized controlled trials. Nephrol Dialysis Transplantation. 2007;22(7):1933–42. 10.1093/ndt/gfm066 17405792

[10] Li P, Shuker N, Hesselink DA, van Schaik RHN, Zhang X, van Gelder T. Do Asian renal transplant patients need another mycophenolate mofetil dose compared with caucasian or African American patients? Transpl Int. 2014;27(10):994–1004. 24963914 10.1111/tri.12382

[11] van Gelder T, Hesselink DA. Mycophenolate revisited. Transpl Int. 2015;28(5):508–15. 25758949 10.1111/tri.12554

[12] Kittanamongkolchai W, Rukrung C, Supasiri T, Lertjirachai I, Somparn P, Chariyavilaskul P, et al. Therapeutic drug monitoring of mycophenolate mofetil for the treatment of severely active lupus nephritis. Lupus. 2013;22(7):727–32. 23651860 10.1177/0961203313486949

[13] Neumann I, Fuhrmann H, Fang IF, Jaeger A, Bayer P, Kovarik J. Association between mycophenolic acid 12-h trough levels and clinical endpoints in patients with autoimmune disease on mycophenolate mofetil. Nephrol Dialysis Transplantation. 2008;23(11):3514–20. 10.1093/ndt/gfn360 18586766

[14] Pourafshar N, Karimi A, Wen X, Sobel E, Pourafshar S, Agrawal N, et al. The utility of trough mycophenolic acid levels for the management of lupus nephritis. Nephrol Dialysis Transplantation. 2019;34(1):83–9. 10.1093/ndt/gfy026 PMC6657446 29548021

[15] Yap DYH, Tam CH, Yung S, Wong S, Tang CSO, Mok TMY, et al. Pharmacokinetics and pharmacogenomics of mycophenolic acid and its clinical correlations in maintenance immunosuppression for lupus nephritis. Nephrol Dialysis Transplantation. 2020;35(5):810–8. 10.1093/ndt/gfy284 30215770

[16] Vu D, Tellez-Corrales E, Yang J, Qazi Y, Shah T, Naraghi R, et al. Genetic polymorphisms of UGT1A8, UGT1A9 and HNF-1α and gastrointestinal symptoms in renal transplant recipients taking mycophenolic acid. Transpl Immunol. 2013;29(1–4):155–61. 23721685 10.1016/j.trim.2013.05.005

[17] Merrigan SD, Kish-Trier E, Seegmiller JC, Johnson-Davis KL. LC–MS/MS method for quantitation of mycophenolic acid, mycophenolic acid acyl-glucuronide, and 7-O-mycophenolic acid glucuronide in serum. Clin Mass Spectrom. 2017;3:41–8. 39193102 10.1016/j.clinms.2017.07.001 PMC11322753

[18] Gladman DD, Ibaņez D, Urowitz MB. Systemic lupus erythematosus disease activity index 2000. J Rhuematol. 2002;29(2):288–91. 11838846

[19] Yee C-S, Farewell VT, Isenberg DA, Griffiths B, Teh L-S, Bruce IN, et al. The use of systemic Lupus Erythematosus Disease Activity Index-2000 to define active disease and minimal clinically meaningful change based on data from a large cohort of systemic lupus erythematosus patients. Rheumatology. 2011;50(5):982–8. 21245073 10.1093/rheumatology/keq376 PMC3077910

[20] Rissling O, Bauer S, Shipkova M, Glander P, Mai M, Hambach P, et al. Simultaneous determination of mycophenolate and its metabolite mycophenolate-7-o-glucuronide with an isocratic HPLC-UV-based method in human plasma and stability evaluation. Scand J Clin Lab Investig. 2016;76(8):612–9. 27676419 10.1080/00365513.2016.1230775

[21] Shaw LM, Holt DW, Oellerich M, Meiser B, van Gelder T. Current issues in therapeutic drug monitoring of mycophenolic acid: report of a roundtable discussion. Ther Drug Monit. 2001;23(4):305–15. 11477311 10.1097/00007691-200108000-00001

[22] Moore RA, Derry S. Systematic review and meta-analysis of randomised trials and cohort studies of mycophenolate mofetil in lupus nephritis. Arthritis Res Therapy. 2006;8:1–10. 10.1186/ar2093 PMC1794528 17163990

[23] Schijvens AM, Ehren R, Weber LT, Schreuder MF. The quest for optimal control of relapses in children with nephrotic syndrome. Kidney Int. 2019;95(3):717. 30784663 10.1016/j.kint.2019.01.002

[24] Kirubakaran N, Punnen A, Prabha R, Agarwal I, Kumar S. Therapeutic drug monitoring of mycophenolate mofetil for the treatment of pediatric lupus nephritis: a cross-sectional study. Indian J Rheumatol. 2022;17(2):124–8.

[25] Ehren R, Schijvens AM, Hackl A, Schreuder MF, Weber LT. Therapeutic drug monitoring of mycophenolate mofetil in pediatric patients: novel techniques and current opinion. Expert Opin Drug Metab Toxicol. 2021;17(2):201–13. 33107768 10.1080/17425255.2021.1843633

[26] Kuypers DRJ, Le Meur Y, Cantarovich M, Tredger MJ, Tett SE, Cattaneo D, et al. Consensus report on therapeutic drug monitoring of mycophenolic acid in solid organ transplantation. Clin J Am Soc Nephrol. 2010;5(2):341–58. 20056756 10.2215/CJN.07111009

[27] Fu L, Huang Z, Song T, He S, Zeng D, Rao Z, et al. Short-term therapeutic drug monitoring of mycophenolic acid reduces infection: a prospective, single‐center cohort study in Chinese living‐related kidney transplantation. Transpl Infect Disease. 2014;16(5):760–6. 25092411 10.1111/tid.12275

[28] Borni-Duval C, Caillard S, Olagne J, Perrin P, Braun-Parvez L, Heibel F, et al. Risk factors for BK virus infection in the era of therapeutic drug monitoring. Transplantation. 2013;95(12):1498–505. 23778568 10.1097/TP.0b013e3182921995

[29] Sagcal-Gironella ACP, Fukuda T, Wiers K, Cox S, Nelson S, Dina B, et al. editors. Pharmacokinetics and pharmacodynamics of mycophenolic acid and their relation to response to therapy of childhood-onset systemic lupus erythematosus2011: Elsevier. 10.1016/j.semarthrit.2010.05.007 PMC3021770 20655577

[30] Sobiak J, Resztak M, Pawiński T, Żero P, Ostalska-Nowicka D, Zachwieja J, et al. Limited sampling strategy to predict mycophenolic acid area under the curve in pediatric patients with nephrotic syndrome: a retrospective cohort study. Eur J Clin Pharmacol. 2019;75:1249–59. 31172249 10.1007/s00228-019-02701-5

[31] Le Meur Y, Büchler M, Thierry A, Caillard S, Villemain F, Lavaud S, et al. Individualized mycophenolate mofetil dosing based on drug exposure significantly improves patient outcomes after renal transplantation. Am J Transplant. 2007;7(11):2496–503. 17908276 10.1111/j.1600-6143.2007.01983.x

