# Supplementary material for: Mycophenolic acid trough level assessment in patients with lupus nephritis; does it make a difference?
Source: Pediatr Rheumatol Online J. 2025 Mar 12;23:26. doi: 10.1186/s12969-025-01074-7 (PMC11905544; doi:10.1186/s12969-025-01074-7)
Supplement: Supplementary file 1 — Supplementary Material 1 [file 12969_2025_1074_MOESM1_ESM.docx]

**Supplementary materials**

**Supplementary figure 1**


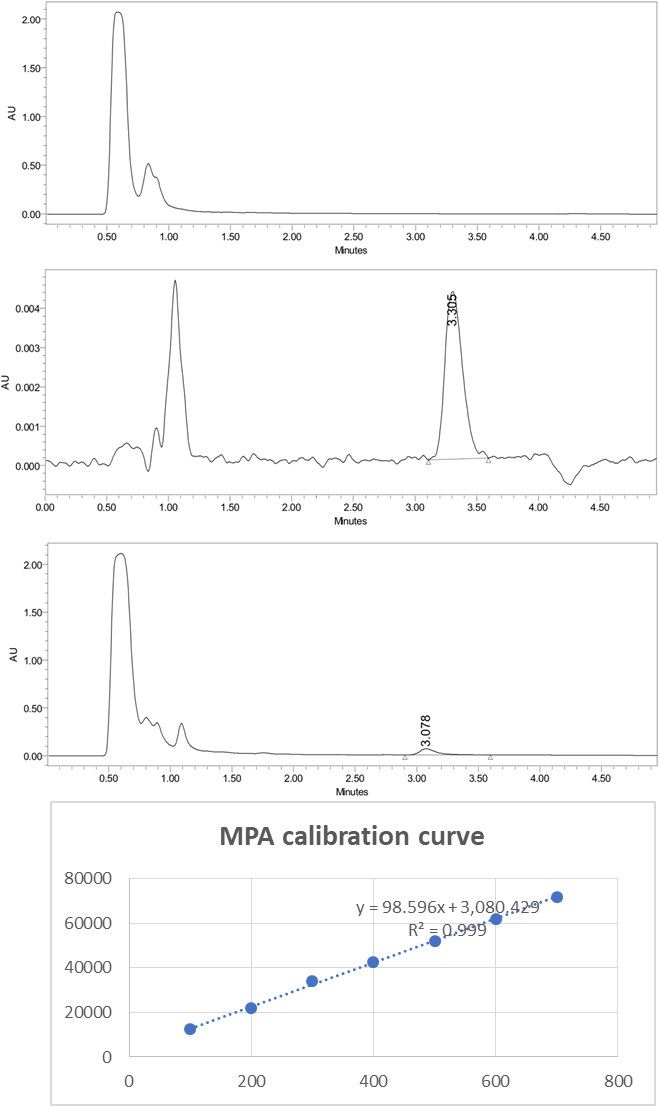


A

B

D

C

**Supplementary figure 1:** (A) Drug-free plasma of a healthy volunteer. (B) 0.4 µg/mL calibration standard (C) Plasma of a patient undergoing maintenance MFF treatment. (D) MPA calibration curve.

**Supplementary figure 2:**

| **< 18 y** |  |  |  |  |  |  |
| --- | --- | --- | --- | --- | --- | --- |
|  | **Area** | **SEM** | **p** | **95% Confidence Interval** | | **Cut off value** |
| **Gastritis** | 0.414 | 0.14 | 0.507 | 0.14 | 0.688 | **0.971 (43% sensitivity & 65% specificity)** |
| **Infection** | 0.231 | 0.085 | 0.369 | 0.063 | 0.398 | **1.29 (60% sensitivity & 71% specificity)** |
| **Flare** | 0.821 | 0.084 | <0.005* | 0.657 | 0.985 | **0.716 (69% sensitivity & 91% specificity)** |
| **M rash** | 0.746 | 0.094 | 0.071 | 0.563 | 0.929 | **0.65 (62% sensitivity & 89% specificity)** |

| **>18 y** |  |  |  |  |  |  |
| --- | --- | --- | --- | --- | --- | --- |
|  | **Area** | **SEM** | **p** | **95% Confidence Interval** | | **Cut off value** |
| **Gastritis** | 0.815 | 0.092 | 0.004* | 0.635 | 0.994 | **1.29 (80% sensitivity & 83% specificity)** |
| **Infection** | 0.708 | 0.112 | 0.181 | 0.485 | 0.932 | **1.04 (75% sensitivity & 63% specificity)** |
| **Flare** | 0.777 | 0.01 | <0.01* | 0.622 | 0.931 | **0.67 (70% sensitivity & 82% specificity)** |
| **M rash** | 0.731 | 0.105 | 0.042* | 0.525 | 0.937 | **0.67 (68% sensitivity & 93% specificity)** |

**Supplementary figure (2):** Receiver operating characteristic (ROC) curves of MPA trough concentration to predict therapeutic outcomes of MFF treatment. The fraction of true-positive (sensitivity) and of false-positive results (1-specificity) for Gastritis (A), infection (B), flare (C), malar rash (D) in pediatrics and for Gastritis (E), infection (F), flare (G), malar rash (H) in adults. A value of 0.5 (reference red line) is no better than by chance and a value of 1.0 reflects a perfect indicator.


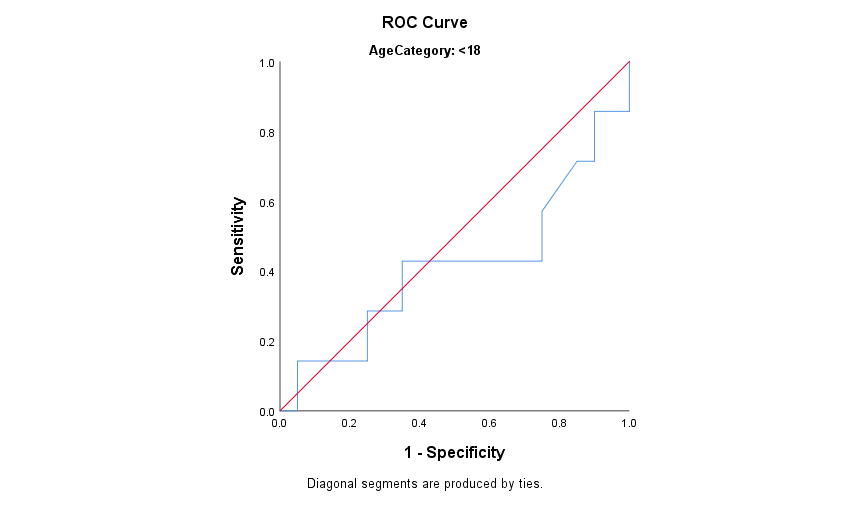

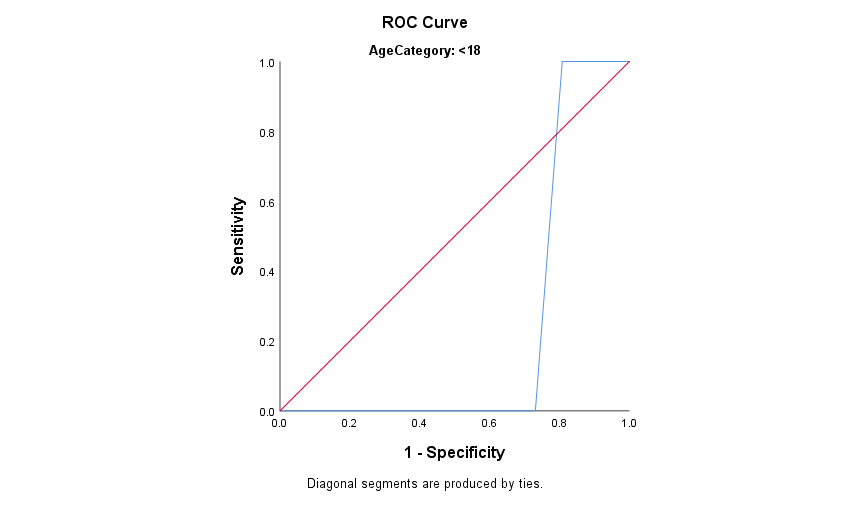

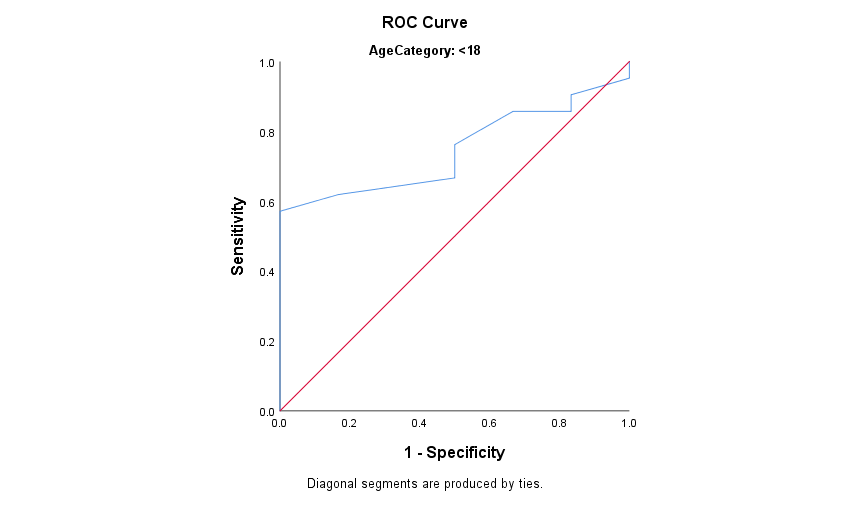

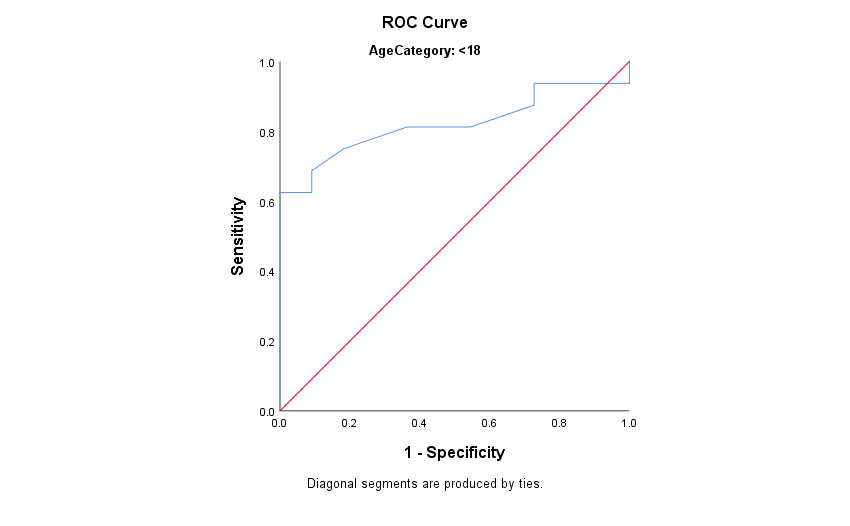

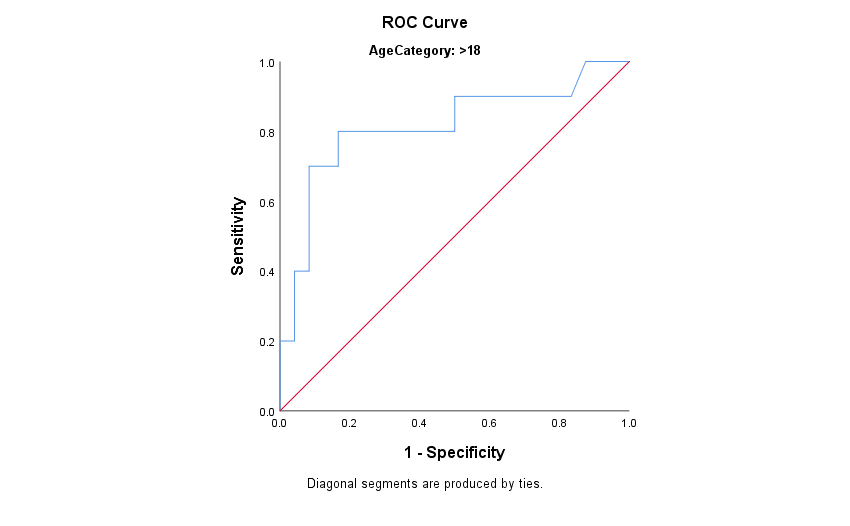

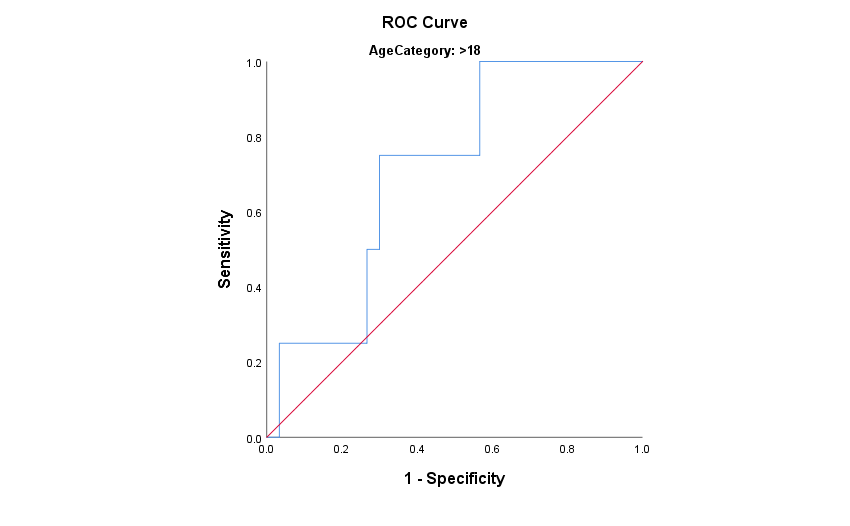

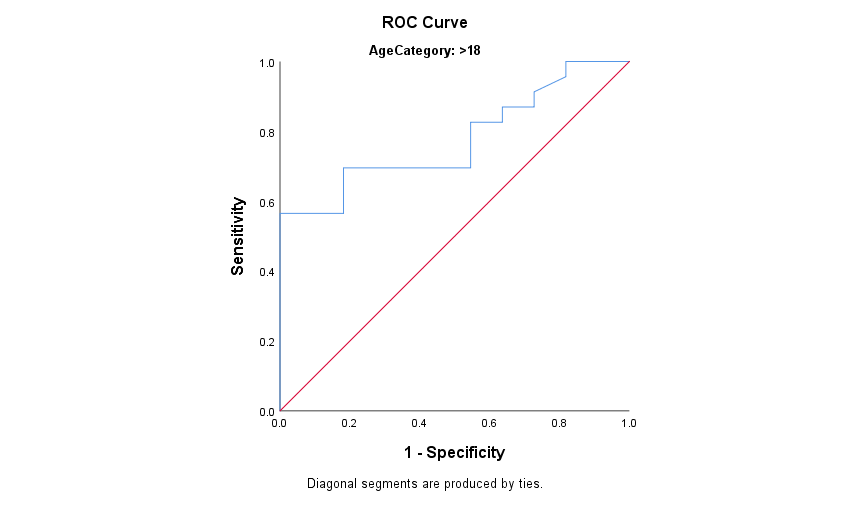

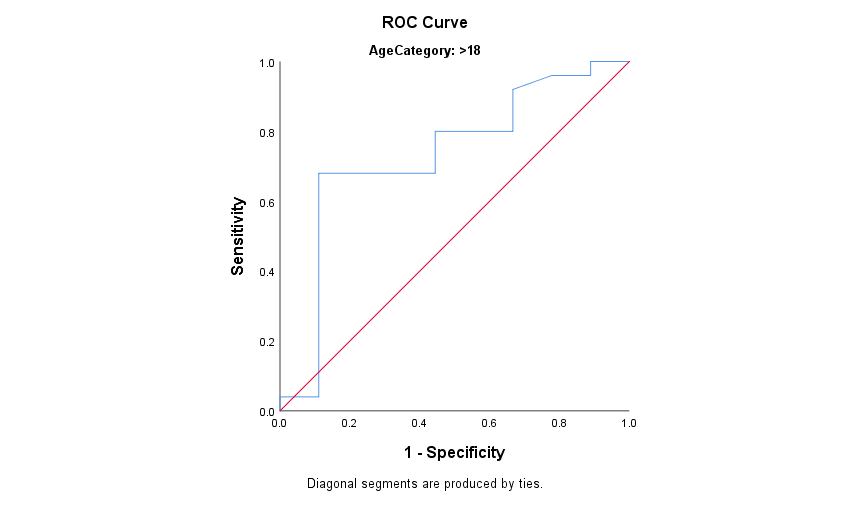


A

B

C

D

E

F

H

G
